# The Quest for a Neurobehavioral Profile of Heavy Prenatal Alcohol Exposure

**Published:** 2011

**Authors:** Sarah N. Mattson, Edward P. Riley

The devastating consequences of fetal alcohol syndrome (FAS) are well established, and, as a leading cause of intellectual disabilities (Pulsifer 1996), FAS has significant societal and public health implications. Importantly, FAS is associated with a broad range of neurobehavioral deficits (for more information, see the article by Coles, pp. 42–50 in this issue). However, FAS is only the most serious possible consequence of heavy prenatal alcohol exposure, and many individuals who do not meet diagnostic criteria for FAS also are severely impacted by gestational alcohol exposure. The term fetal alcohol spectrum disorders (FASD) is used to encompass a spectrum of effects that includes FAS towards the extreme end as well as conditions such as alcohol-related neurodevelopmental disorder (ARND) and alcohol-related birth defects (ARBD). The term ARND (Hoyme et al. 2005) refers to individuals who, after heavy prenatal alcohol exposure, exhibit neurobehavioral effects without meeting the physical criteria for FAS (for a review, see Vaurio et al. 2010). Clinical identification of this group of individuals is hampered precisely because they do not exhibit the external physical features of FAS, and the existing physiological biomarkers for gestational alcohol exposure have several limitations. Determination of a profile based on the neurobehavioral effects of heavy prenatal alcohol exposure would allow more accurate identification of affected individuals. To be clear, development of such a profile is aimed at identifying and characterizing those who are affected by prenatal alcohol exposure, not simply those who have been exposed to alcohol prenatally.

## Challenges of Establishing a Neurobehavioral Profile of Heavy Prenatal Alcohol Exposure

Although the main goal of the quest for a neurobehavioral profile of heavy prenatal alcohol exposure is improved identification of alcohol-affected individuals, additional benefits include enhanced intervention and treatment opportunities for these individuals as well as improved accuracy of incidence estimates. Thus far, accurate identification of individuals who are affected by alcohol exposure has proven difficult for several reasons. First, clinicians cannot rely only on external markers because the majority of alcohol-affected people do not meet the physical criteria for FAS. Second, the full range of effects of prenatal alcohol exposure is not currently known; therefore, individuals with less striking or atypical manifestations may be misdiagnosed or fail to get a diagnosis altogether. Third, individual neurobehavioral features, including decreases in an individual’s intelligence quotient (IQ), also may result from clinical conditions or disorders other than FASD, further decreasing the ability to accurately identify individuals as alcohol affected. Finally, individual differences in factors that influence the consequences of prenatal alcohol exposure, such as the dose or timing of exposure, genetic variability, nutritional status, or postnatal factors, might interfere with developing a unifying neurobehavioral profile.

There currently are two main avenues for identifying individuals with prenatal alcohol exposure: maternal reports of drinking in pregnancy and dysmorphology exams. Maternal reports of exposure, if accurate, are ideal for identifying people who have been exposed to alcohol prenatally. Such data can be collected during pregnancy (i.e., prospectively) or afterwards (i.e., retrospectively). Recent research suggests that compared with prospective reports, retrospective maternal reports of gestational alcohol use indicate higher levels of exposure and are better predictors of behavioral outcomes in teenagers with histories of prenatal alcohol exposure (Hannigan et al. 2010). However, accurate maternal reports are notoriously difficult to obtain retrospectively because a large proportion of individuals with heavy prenatal alcohol exposure who participate in research studies are in foster or adoptive care (Streissguth et al. 2004). Accordingly, many research studies that involve retrospectively-collected alcohol exposure data rely on other sources, such as social service, medical, legal, or adoption records. This may lead to a form of selection bias insofar as only the most blatant cases of prenatal alcohol exposure typically are documented. However, these cases also are representative of the population of individuals who are of greatest clinical concern—that is, those with heavy prenatal alcohol exposure who are experiencing behavioral, neuropsychological, and psychiatric disturbances.

The second avenue for identifying people with prenatal alcohol exposure—the analysis of altered morphological features (i.e., dysmorphology examination)—is standard practice for identifying affected individuals. This examination typically documents physical markers associated with exposure to substances that can harm the embryo (i.e., teratogens) such as alcohol, or with other genetic or medical conditions. The physical markers of alcohol exposure are well documented and include short palpebral fissures, a smooth philtrum, a thin vermillion border, and small brain size (i.e., microcephaly). (For more information, see the article by Warren and colleagues, pp. 4–14.) Although the combination of these markers is relatively specific to prenatal alcohol exposure and the diagnosis of FAS can be made even if the person’s history of prenatal alcohol exposure is unknown, it is not sufficiently sensitive to detect all affected individuals because not all affected persons show the physical features associated with FAS. In fact, the number of nondysmorphic individuals born to heavy-drinking women likely is at least three times greater than the number of cases of FAS (Bertrand et al. 2005; Sampson et al. 1997). This difference in prevalence may be because the facial anomalies associated with FAS are thought to result from exposure during a fairly limited time during the first trimester of pregnancy, whereas brain anomalies could arise from exposure anytime during gestation.

A third potential avenue for identifying alcohol-exposed people is through detection of long-lasting alcohol metabolites. The most widely studied putative biological biomarker is fatty acid ethyl esters (FAEEs) in the meconium^1^ of newborns. Researchers have suggested that FAEE measurement can be a sensitive and specific mechanism for detecting infants that have been exposed to alcohol during the latter half of pregnancy (e.g., Ostrea et al. 2006). The identification of such a biological biomarker would be advantageous (Burd and Hofer 2008), and the promise of improving detection of alcohol-exposed infants even has led to a call for universal meconium screening (Hutson et al. 2010). Other researchers (Burd and Hofer 2008; Lester et al. 2001), however, have suggested caution for both methodological and ethical reasons. Furthermore, although FAEE quantification in meconium may prove useful in identifying alcohol-exposed newborns, it has several limitations. First, it is not useful for detecting exposure that occurred during the first trimester. Second, it does not allow identification after the newborn period unless samples are appropriately stored. Finally, FAEE is a marker only of alcohol exposure but not of alcohol’s effects and therefore may be too inclusive. More recent research is examining the possibility of measuring FAEEs in maternal hair as amarker of exposure during various periods of pregnancy, although this still is in experimental stages.

Consequently, development of a neurobehavioral profile that characterizes the effects of heavy prenatal alcohol exposure in affected individuals regardless of the presence of physical features (and therefore the diagnosis of FAS) has the potential to overcome the limitations of the aforementioned methods of identifying alcohol-affected individuals. It also would allow a more accurate assessment of the neurobehavioral effects across the spectrum of FASD.

The quest for a disorder-specific neurobehavioral profile is not without precedent. For example, the understanding of Williams syndrome^2^ has been enhanced by the characterization of behavioral and cognitive strengths and weaknesses of affected individuals (Bellugi et al. 1990; Mervis et al. 2000). However, identification of such a neurodevelopmental profile may be even more important in disorders like those encompassed by FASD for which no physiological markers or clear diagnostic criteria exist. For example, although disorders such as autism or attention-deficit/hyperactivity disorder (ADHD) lack specific biological markers, diagnostic criteria have been have identified (American Psychiatric Association 2000). Alternatively, genetic disorders such as Down syndrome or Williams syndrome have a clear genetic etiology and therefore can be identified by determining the individual’s genetic makeup (i.e., genotype). In such cases, neurobehavioral profiles can help to improve understanding of the disorder and develop rational intervention and treatment programs. For example, treatments could be developed that specifically target areas of weakness while exploiting areas of relative strength. Conversely, for alcohol-affected individuals, a neurobehavioral profile has the dual purpose of improving both identification and treatment. For example, if remediation targeted memory impairment, it would be beneficial to understand that children with FASD have greater problems with learning than with recall of learned information and therefore may benefit from repeated exposure to new information (Mattson and Roebuck 2002).

## Current Approaches to Establishing a Neurodevelopmental Profile

To provide all these benefits, a neurobehavioral profile associated with heavy prenatal alcohol exposure must be both sensitive (i.e., must correctly identify alcohol-affected individuals) and specific (i.e., correctly exclude individuals who are not alcohol affected). Identification of a profile with good sensitivity is fairly straightforward in principle; it involves identifying a cognitive ability or set of abilities that are deficient in alcohol-affected individuals. Nearly four decades of research have detailed numerous neurobehavioral deficits in alcohol-affected individuals (for are view, see Vaurio et al. 2010), and recent research suggests that executive function and spatial processing are especially sensitive to prenatal alcohol exposure (Mattsonet al. 2010). Nevertheless, a useful neurobehavioral profile has been elusive becauseit is more difficult to determine which individual deficits, or which pattern of deficits, is specific to this population. For example, low IQ scores occur in many (but not all) alcohol-affected individuals but also in many other developmental disorders. Thus, this important aspect of functioning is neither sensitive (because not all alcohol-affected individuals have low IQ scores) nor specific (because many other individuals without prenatal alcohol exposure have low IQ scores).

In an effort to address the question of specificity, researchers in recent years have compared alcohol-affected children with other groups of children who exhibit certain traits (i.e., phenotypic aspects) similar to alcohol-affected individuals. These comparisons can help identify characteristics that differ between groups and therefore are specific to alcohol-affected individuals. Thus far, these efforts have focused on two primary effects of heavy prenatal alcohol exposure: low IQ score and a diagnosis of ADHD. When children with heavy prenatal alcohol exposure are compared with contrast groups with similar IQ scores, both similarities and differences emerge. Similarities (i.e., non-specific findings) include performance on measures of internalizing behavior (Mattson and Riley 2000), expressive and receptive language ability (McGee et al. 2009), sustained attention, and retention of verbal material (Vaurio et al. 2011). In contrast, alcohol-affected children are more impaired than IQ-matched control subjects on measures of externalizing behavior (Mattson and Riley 2000), adaptive skills (Thomas et al. 1998; Whaley et al. 2001), and verbal learning (Coles et al. 2010; Mattson et al. 1996; Vaurio et al. 2011), and these measures therefore can be considered specific findings. These observations suggest that in comparison to other groups with lowered IQ scores, it is possible to identify a profile of effects that are specific to prenatal alcohol exposure.

Other research has documented both similarities and differences between children with heavy prenatal alcohol exposure and nonexposed children with ADHD. These studies found that some aspects of executive function may prove useful in profile development because deficits in this domain are not entirely overlapping in these two groups. For example, children with ADHD or FASD show deficits on measures of nonverbal problem solving and worse letter fluency than category fluency, but only alcohol-affected children demonstrate greater impairment on letter fluency and set-switching (Vaurio et al. 2008). Other studies have documented greater impairment in alcohol-affected children than nonexposed children with ADHD on measures of visual-spatial reasoning, problem solving, flexibility, and encoding and shift aspects of attention (Coles et al. 1997), as well as in social cognition and facial emotion processing ability (Greenbaum et al. 2009). Differences in adaptive skills also have been noted, with alcohol-affected children demonstrating an arrest indevelopment of adaptive ability (Crocker et al. 2009; Thomas et al. 1998; Whaley et al. 2001), whereas nonexposed children with ADHD demonstrate delayed ability that improves with age (Crocker et al. 2009). Finally, differences in classical conditioning exist between children with FASD and children with ADHD (Coffin et al. 2005). Thus, although the rates of ADHD are high in alcohol-affected children, the presence of this comorbidity does not account for some of the neurocognitive deficits in this population.

One weakness of this approach of comparing alcohol-affected children with other clinical contrast groups (e.g., nonexposed children with low IQ or ADHD) is that these studies have proceeded, by necessity, in a piecemeal fashion, and no one study can simultaneously account for all aspects of the alcohol phenotype. To date, only two pieces of the FASD puzzle have been addressed, and additional research addressing other potential contributory factors or including other comparisons is needed. Another important consideration in the development of a neurobehavioral profile for individuals affected by heavy prenatal alcohol exposure is the possibility that more than one profile may exist. For example, differences in the patterns (i.e., binge drinking versus more continuous alcohol consumption) or timing of alcohol exposure (e.g., O’Callaghan et al. 2007) may result in different profiles. Moreover, the profile may be affected by other factors such as overall IQ score (i.e., there may be different profiles for children with average versus below-average IQ scores) (e.g., Carmichael Olson et al. 1998; Kerns et al. 1997) or age of the children (e.g., Crocker et al. 2009). It also is possible that other environmental or genetic factors may interact with exposure, resulting in multiple profiles. Hopefully, however, even these profiles will share a core set of features that can be used to accurately identify individuals affected by heavy prenatal alcohol exposure.

## Conclusion

The quest for a neurobehavioral profile of heavy prenatal alcohol exposure is essential for enhancing understanding of the effectsofthis exposure and will result in improvements in intervention and incidence estimates. Although the search already has been fruitful, a reliable profile has been elusive. Additional research is necessary to further clarify the sensitivity and specificity of the emerging profile as well as to continue to address the confounding effects of myriad complicating factors.

## Footnotes

1 Meconium is the earliest stool passed by an infant and is composed of materials ingested while the infant was in the uterus.

2 Williams syndrome is a rare, genetically determined neurodevelopmental disorder characterized by mental disability, heart defects, and unusual facial features (i.e., by a distinctive “elfin” facial appearance).
